# Editorial: Disparate roles of mitochondria in cell survival and cell death: new insight from the CNS

**DOI:** 10.3389/fnins.2023.1325250

**Published:** 2023-11-15

**Authors:** Raghu R. Krishnamoorthy

**Affiliations:** Department of Pharmacology and Neuroscience, North Texas Eye Research Institute, University of North Texas Health Science Center, Fort Worth, TX, United States

**Keywords:** mitochondria, neurodegeneration, fusion, fission, mitophagy

There has been a resurgence in research on the role of mitochondria, particularly in the field of neurodegeneration. Three decades ago, there was a profusion of publications addressing detailed mitochondrial mechanisms contributing to cell death via the caspase family of proteases and the role of Bcl2 family of proteins that acted as either pro- or anti-apoptotic agents influencing cell death. In recent years, research on mitochondria has taken forays into new directions, including, mitochondrial fission, fusion, biogenesis and mitophagy. It is becoming increasingly evident that these processes are fine-tuned and carefully regulated to maintain bioenergetics and mitochondrial dynamics in various cell types, particularly excitable cells such as neurons and muscle cells. The central nervous system, including the brain and the retina have high energy requirement and oxygen consumption, hence, are characterized by an abundance of mitochondria. Hence while mitochondria are present in all tissues in the body, the impact of mitochondrial gene mutation is felt disproportionately in the brain and the retina and exemplified by conditions such as Parkinson's disease, Alzheimer's disease, Leber's Hereditary Optic Neuropathy and Leigh syndrome. These mitochondrial findings have evoked great interest in mitochondrial pathways contributing to the etiology of these neurodegenerative diseases.

Atkinson et al. provide a synopsis of mitochondrial modifications and change in mitochondrial dynamics that occur in the demyelinating disease, multiple sclerosis. Since a loss of myelin results in a decline in salutatory conductance, this produces an increased energy demand for axons to maintain resting potential and axonal function, leading to changes in mitochondrial morphology, and expression of components of the electron transport chain. The authors discuss current approaches to study mitochondrial structure, transport and function and recently developed methodologies to study mitochondria-specific gene expression and transgenic mouse lines that express Cre recombinase driven by CNS specific promoters to study mitochondria in specific cell populations in the CNS.

Makarov et al. discuss the diverse roles of presenilin in contributing to neurodegeneration in Alzheimer's disease (AD). While it is known that mutations in presenilins result in formation of amyloidogenic Aβ42 and neurodegeneration in familial Alzheimer's disease, findings from animal models of AD point to other etiological mechanisms, including, calcium dysregulation, mitochondrial dysfunction, compromise of mitophagy and autophagy. The review discusses the effect of presenilins on mitochondrial dynamics, axonal transport, mitochondrial fusion and fission. The effect of presenilin mutations on mitochondria associated ER membranes (MAMs), ER-mitochondria cross talk, and calcium homeostasis are also discussed.

Johnson et al. address the topic of mitochondrial calcium channels, including, mitochondria calcium uniporters (MCU), Na^+^/Ca^2+^ exchangers (NCX) and transient potential melastatin 2 (TRPM2), as key players in regulating mitochondrial calcium. The authors also describe the involvement of voltage-dependent anion channels (VDACs) in calcium uptake into the mitochondria and its role in calcium homeostasis and cell death. Dysregulation of calcium signaling in Alzheimer's disease, Parkinson's disease and Huntington's disease and potential strategies for neuroprotection through NCX expression or MCU inhibition are discussed.

The concept of mitochondria- associated ER membranes (MAMs) is gaining traction in the light of several findings of a cross talk between the mitochondria and ER. Pham et al. discuss different aspects of mitochondrial and ER dysfunction, particularly the unfolded protein response (UPR) and the involvement of MAMs in coordinating their crosstalk, from the perspective of glaucomatous neurodegeneration. The authors describe various protein components of MAMs, including VDAC and inositol 1, 4, 5 trisphosphate receptor (IP3 receptor), which facilitate calcium uptake into the mitochondria. The role of MAMs in regulating calcium dynamics and retinal ganglion cell death is also discussed.

Damaged mitochondria are enclosed by a double-membraned structure called phagophore, which mature into autophagosome that fuse with lysosome to target the damaged mitochondria for degradation. This process is important as a quality control mechanism for cells to retain healthy mitochondria, which in turn is necessary for optimum health of tissues and the organism. A decline in mitophagy has been reported to be a contributor to neurodegenerative diseases, including, Alzheimer's disease and Parkinson's disease. Brooks et al. discuss the involvement of mitophagy in three ocular neurodegenerative diseases, namely, diabetic retinopathy, glaucoma and age-related macular degeneration (AMD). The precise role of mitophagy in ocular neurodegeneration is still being actively investigated; however, several animal models of ocular neurodegenerative diseases suggest that a decline in mitophagy could be a contributor to neurodegeneration. There are also some findings that suggest an increase in mitophagy in some animal models of glaucoma. The authors point out that discrepancy in these findings is possible due to differences in the animal models of the disease, methodologies and techniques used to assess mitophagy.

It is clear that new insights on mitochondrial morphology, bioenergetics, dynamics, and mitophagy are providing leads to unravel hitherto unclear mechanisms underlying neurodegeneration in several brain and ocular neurodegenerative disorders. Developments in cell biology tools and imaging technologies are providing unprecedented level of image resolution and understanding of cell function, which could help elucidate cellular and molecular mechanisms of neurodegeneration and aid in development of neuroprotective approaches to treat these challenging diseases.

## Author contributions

RK: Conceptualization, Validation, Writing – original draft, Writing – review & editing.

